# Using Geocaching to Promote Active Aging: Qualitative Study

**DOI:** 10.2196/15339

**Published:** 2020-06-11

**Authors:** Silvia Fornasini, Marco Dianti, Alessandro Bacchiega, Stefano Forti, Diego Conforti

**Affiliations:** 1 Fondazione Bruno Kessler Trento Italy; 2 Department of Psychology and Cognitive Science University of Trento Trento Italy; 3 TrentinoSalute 4.0 Trento Italy

**Keywords:** geocaching, active ageing, elderly empowerment, qualitative methods

## Abstract

**Background:**

Over the past few years, the development of technologies supporting active aging has been increasing. Among the activities that promote physical exercise by using technologies is geocaching—a treasure hunt of sorts in which participants use a receiver GPS to hide or find real or virtual objects. Although this activity is particularly suited to the promotion of healthy lifestyles in older people, geocaching remains to be unexplored in this area.

**Objective:**

This study aimed to investigate the effectiveness of activities combining geocaching and self-tracking technologies to promote active aging, evaluate the usability of technologies, and explore the ways in which technologies have been integrated in the organizational context under examination to determine the user experience of participants.

**Methods:**

A group of individuals aged 65 years and older (N=14) attending a senior center managed by a social cooperative was involved in the study. Some of them created the geocaching contents, and others, split into 2 teams, participated in the game. Each participant was given a pedometer bracelet and the geocaching app. The steps taken by individual participants along with the number of caches found by each group translated into team scores.

**Results:**

The main results of the study were as follows: (1) activities in favor of active aging that involve the use of new technologies can foster the participation of elderly people; in particular, adding gamification to self-tracking can be a valid strategy to promote physical exercise among the elderly; (2) for this to happen, involvement of older people firsthand is crucial, and there must be a focus on their active involvement and empowerment in every phase of the project; and (3) the mediation of conflicts and competition that arise from the gamification could only take place because of the strong support of the organization in the form of social workers.

**Conclusions:**

The results show that promoting active aging through technologies requires more effort than simply using these tools; it requires a wider process that involves an articulated organizational network with heterogeneous actors, technologies, and relations.

## Introduction

### Challenging Aging Through Technology

Population aging involves an increase in the demand for care and an overload of the pension and welfare system. For this reason, aging can be considered a challenge for society [1]. To overcome this challenge, the promotion of active aging is essential, and one key strategy is the promotion of physical exercise. Regular physical activity delays functional decline, reduces the risk of chronic diseases and falls, improves the quality of life, and helps elderly people remain independent for as long as possible [1,2]. Over the past few years, the development of technologies supporting physical activity has been increasing, and the effectiveness of these tools in the promotion of active aging has also been demonstrated. Several studies have underlined the beneficial impact of technologies on the daily lives of this segment of the population. Technologies allow for an increase in physical activity [3,4] and combat loneliness by increasing sociality, self-esteem, and cognitive functions in the elderly [5]. With regard to the type of technologies used, the literature is divided into 2 main strands: on the one hand, studies that deal with devices for self-tracking of physical exercise, such as pedometer apps and wristbands [6,7], and, on the other hand, a recent trend has revealed virtual physical training through exergames [8,9] and serious games [10,11], making gamification an effective tool to motivate older people to exercise.

### From Self-Tracking to Gamification

The most recommended physical activity for the elderly is walking. Independence in a person’s life is strongly based on the ability to walk, an activity that allows one to carry out most daily tasks [12]. Emerging persuasive technology and ubiquitous wearable sensors offer much promise for improving health and fitness practices. These technologies can persuade the individual user to change his/her lifestyle and integrate a greater amount of physical activity into their daily life. Persuasive technologies use varied strategies for influencing behavior and activities, most notably self-tracking. Research has made great strides in understanding several aspects of these technologies, such as how their design affects activity and behavior, how to use visualization to motivate activities and provide awareness, and how feedback is understood and used [13,14]. A variety of monitoring devices have been studied and analyzed for their persuasive influence on practices and behavior. One of the most common devices is the pedometer, which is used to measure and motivate physical activity and also to set personal step goals [15]. With regard to elderly people, the literature highlights how technologies allow for an increase in physical activity levels and an improvement in self-esteem and cognitive functions [4,16]. However, some studies have reported that only 26% of these tools are used twice and that 33% of owners abandon the use of sensors after 6 months [17]. To enhance self-tracking mechanisms, gamification strategies have therefore become widespread. These include using rewards and incentives to support users’ motivation and involving users in identifying and establishing their own goals [18-20]. Some studies have reported how the use of motivational elements such as rewards, feedback and progress bars, and competition can encourage older people to use technology and slow down cognitive aging. This highlights the reasons why they adopt, continue, or abandon such practices [21-23]. The literature on how the context of sport helps the elderly negotiate the aging process shows how competition in physical activity helps in redefining aging in terms of physical competency, social engagement, and mental stimulation [24].

Other studies have highlighted the relationship between user motivations and social behavior in the social context of self-tracking technologies [25-27]. For example, a study by Spillers and Asimakopoulos [27] explores how self-tracking is not just meaningful in a rational or an instrumental utilitarian sense but also in the sense of being a source of joy and pleasure for the individual. To account for the meaning of self-tracking, Spillers and Asimakopoulos [27] proposed conceptualizing self-tracking as a social and cultural practice that is fundamentally communicative. The combination of competition, communication, and cooperation consistently led to higher levels of intrinsic motivation [28,29].

### Geocaching for Active Aging

Among the activities that promote physical exercise by using technologies, geocaching, a treasure hunt of sorts in which participants, called *geocachers*, use a receiver GPS to hide or find real or virtual objects (called caches), is one of them. Several studies have highlighted the benefits of this activity for the elderly. Studies that have addressed geocaching from an active aging perspective report the positive effects of spending time outdoors on health, well-being, and sleep [30,31]. Geocaching also stimulates group physical exercise, emphasizing the dimensions of social relations and interactions. It can therefore become a means of communication with family and friends, thus reducing the effects of social isolation that are often involved in aging [31,32]. Other studies have reported how geocaching allows older people to stay informed in an ever-changing environment and share an interest in a city’s historical and artistic heritage [33]. It also provides the opportunity to learn and apply new knowledge in the field of Information and Communications Technology such as the use of digital devices and geolocation [30,34-36]. Geocaching is particularly suited for the promotion of healthy lifestyles in older people, because it combines outdoor activity with new technologies. However, the literature tends to emphasize the social, relational, and recreational aspects of the game, and how geocaching could effectively motivate elderly people to stay active is still unexplored.

### Objectives of the Study

The Impronte project was created in 2017 as part of a series of initiatives aimed at promoting healthy lifestyles by the TrentinoSalute 4.0 Competence Center, which includes the Department of Health and Social Solidarity of the Province of Trento, the Provincial Health Services, and the Bruno Kessler Foundation. This study, which involved the senior center *Contrada Larga* of Trento, Italy, managed by the Kaleidoscopio social cooperative, aimed to show the results of a geocaching activity combined with the use of pedometer bracelets aimed to promote active aging. There were 3 main objectives of the study: first, to investigate how geocaching could effectively motivate elderly people to stay active, in addition with combining a game with the use of self-tracking technologies to quantify physical activity; second, to evaluate the usability of technologies; and third, to explore the ways in which technologies have been integrated into the organizational context under examination to determine the user experience of participants.

## Methods

### Context

To involve participants aged 65 years and older, the service center for elderly people, *Contrada Larga* of Trento city, was contacted. The center is managed by the *Kaleidoscopio* social cooperative that has encouraged activities concerning active aging for years. The service center is considered to be a meeting point for self-sufficient elderly people who use the spaces to meet, find and exchange useful information, have discussions with operators and other people, read newspapers, and use computers. The project activities were therefore fitted into a context that was particularly attentive: care was taken to promote the active participation of the elderly through their involvement in the ideation and realization of the activities. Thanks to the intervention of the center operators, 14 people aged between 65 and 82 years, 13 women and 1 man, were involved (Table 1).

**Table 1:** Demographics of study participants.

Demographics		Number of participants	
**Age (years)**			
	60-70		6
	71-80		7
	Above 80		1
**Gender**			
	Male		1
	Female		13
**Level of education**			
	Primary school		N/A^a^
	Secondary school		3
	Tertiary school		7
	Degree		4

^a^N/A: not applicable.

### Study Design

**Figure 1:**
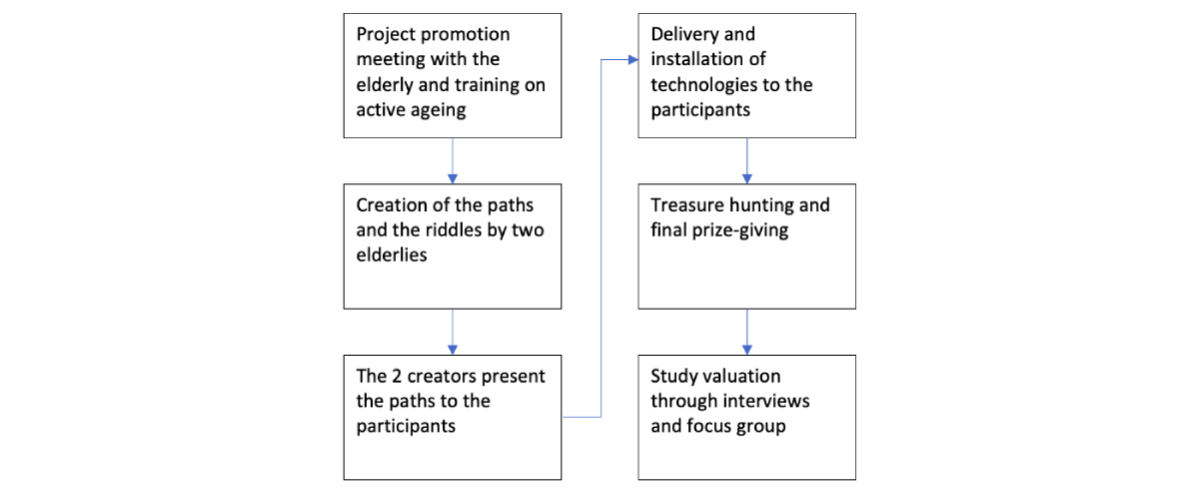
Flowchart of project phases.

The project took place between April and May 2017 and was divided into 6 main phases (Figure 1):

1. First meeting: project activities, methods, and roadmap were presented to the participants. The meeting was an opportunity to offer a training introduction on active aging and on the importance of adopting a healthy lifestyle.
2. Ideation of the treasure hunt by two of the participants who collaborated in the creation of the contents of the game, identifying culturally or historically significant places in the local area.
3. Peer-to-peer presentation of the treasure hunt. A description, by researchers, of the functionalities of the technologies and the score collection mechanism.
4. The delivery of pedometer bracelets to participants, installation of the geocaching app on the participants’ smartphones, team formation, and designation of team captains.
5. Treasure hunt and final distribution of prizes.
6. Study evaluation through interviews and focus groups.

### The System

**Figure 2:**
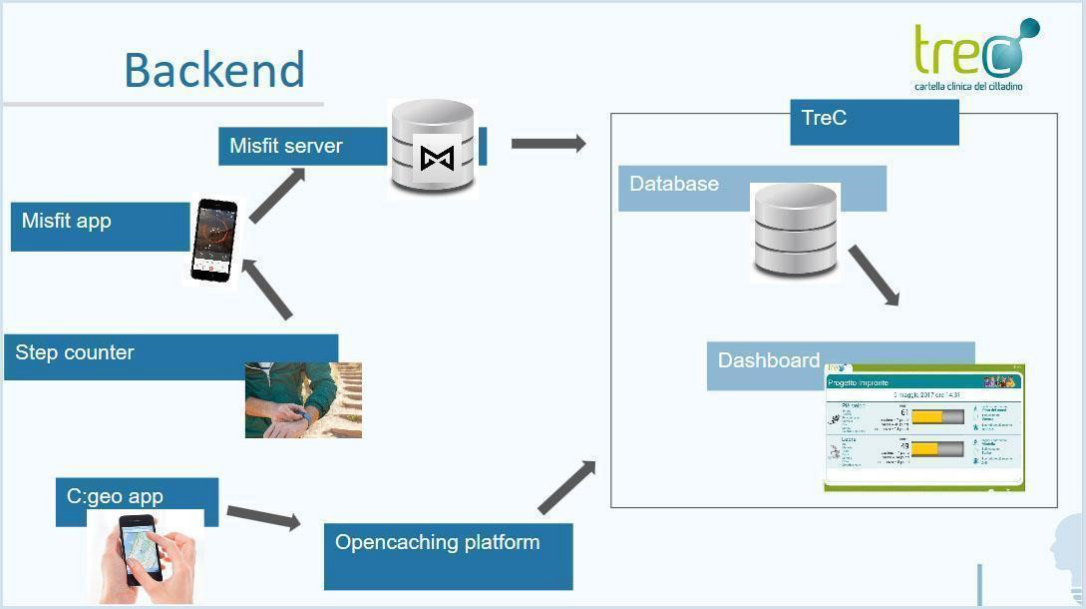
The backend system to collect the steps and the data from the C:Geo app and to show them in the dashboard.

The idea behind the project was to combine the geocaching activity, accompanied by some gamification elements, with the use of fitness tracking technology in the form of a pedometer bracelet. The steps taken by individual participants along with the number of caches found by each group translated into team scores. Each participant had an activity goal of 10,000 steps a day, as advised by the World Health Organization [2]. The project therefore entailed the creation of a system that would connect the technological devices required for geocaching with those necessary for gamification: the opencaching.de website; the C: geo app; and the pedometer bracelet, a digital dashboard for step tracking and reporting of the caches found (Figure 2).

The *opencaching.de* website was selected as we preferred an open-access platform. Unlike other geocaching platforms, it is created by users, allowing anyone to register and play. It also provides the application programming interfaces (APIs) needed to access the data (number of finds, position, comments, etc). The APIs were critical to load the score on the dashboard and keep it updated daily during the challenge. To keep the score of each team, the total number of caches found was included, which we accessed from the opencaching.de website in the caches_found attribute.

The *C: geo app*, available for Android, was selected for the cache search. The app is free and is among the most complete in terms of functionality and compatibility with geocaching services. Being fully compatible with the open caching platform, it was the tool that allowed the teams to read the riddles from the open caching platform, find the caches, log them, and leave comments.

With regard to self-tracking technologies, we chose to give to each participant a pedometer bracelet. The score of each participant as well as that of the team was visible on the digital monitoring dashboard and was automatically calculated based on the number of steps taken, the number of caches found during the outings, and the number of days in which users walked more than 10,000 steps. For both teams, the scores ranged from 0 to 100, calculated according to the following proportions: cache score, 40%; step score, 40%; and constancy score, 20%. The cache score was given by the number of caches found, and the step score was 100 for the team with the highest number of total steps and a proportionally lower number for the other team. The last component of the score, *constancy*, was determined as a proportion between the number of days elapsed since the start of the race and the number of days in which the team members exceeded 10,000 steps. The data were made available periodically to the participants at the senior center or on the dedicated website.

### Building Active Aging in a Participatory Perspective

**Figure 3:**
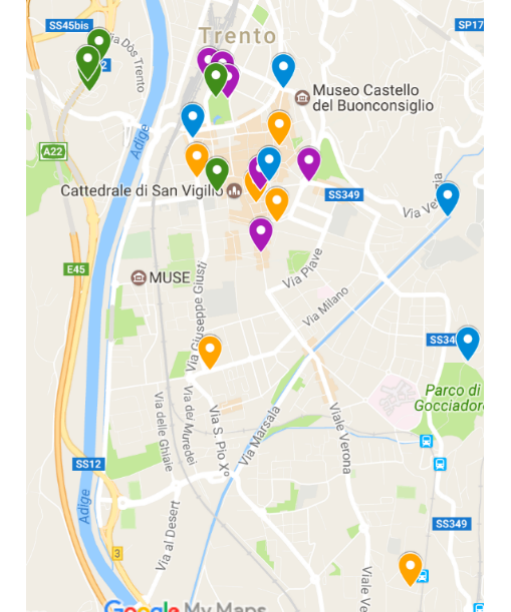
The points of the treasure hunter in the city of Trento.

One of the main characteristics of the project was an approach that would allow the elderly to participate actively in most of the project phases. For this reason, the treasure hunt was created by two of the participants who identified the stages of the hunt and the riddles to be solved without the help of the researchers. The 2 *creators*, after taking a tour of the city center and its surroundings, decided to design a treasure hunt divided into 4 thematic routes: “The city over the centuries,” “The Italian hallmarks of the city,” “Water,” and “Buildings and monuments.” For each route, the 2 creators identified 5 or 6 historically, artistically, and culturally relevant points (Figure 3). For each point, they created a card with its description, some curiosities, and a riddle, which could only be solved by visiting the place. In total, 24 points of interest were designed. The cards were then shared and discussed with the researchers, who uploaded them to the opencaching.de website, thus creating the actual virtual caches. The 2 creators had the task of describing the characteristics of the routes to the group of participants during a meeting that preceded the treasure hunt. During this meeting, the duration, modalities, and characteristics of the routes were presented to the group of seniors, without excessively disclosing the details to keep the curiosity alive. Under the supervision of the center operators, the participants split into 2 teams and appointed 2 team leaders with the task of using the C: geo app during the geocaching activity.

### The Treasure Hunt and Final Awarding

A total of 4 treasure hunts were created, 1 per week. The 2 teams would meet at the senior center half an hour before each hunt to prepare and organize the search, following which the teams would set out on the search to reach the greatest number of stages in the set time. We had to decide whether to encourage learning about technology by giving each participant a smartphone with the app and risk-isolated participation or whether to encourage the social aspect of the game by stimulating communication between participants. We chose the second option, and each team was accompanied by an operator. The senior center was the departure and arrival point for the participants. Both team leaders had a smartphone equipped with the C: geo app and were tasked with guiding the team’s search for the stops.

Before each hunt, we recommended that participants try to leave comments and photographs in the app at each stage. This was done because one of the recreational and relational aspects of geocaching is the possibility to contribute to the contents of the game firsthand. We decided to try to focus the activity also on this aspect because the literature on elderly competition in physical activity shows that the combination of competition and cooperation consistently led to higher levels of motivation. By commenting on the cache and taking pictures, participants would have the opportunity to collaborate more. The 2 creators of the hunt, knowing the solutions to the riddles, were not able to participate in the outings, but they contributed nonetheless to the score of their team—they did not know to which team they had been assigned until the end—by accumulating steps. The final event was an opportunity to sum up the outcomes of the experience as well as to award the prize to the winning team. Each participant was given a certificate of participation.

### The Evaluation

**Figure 4:**
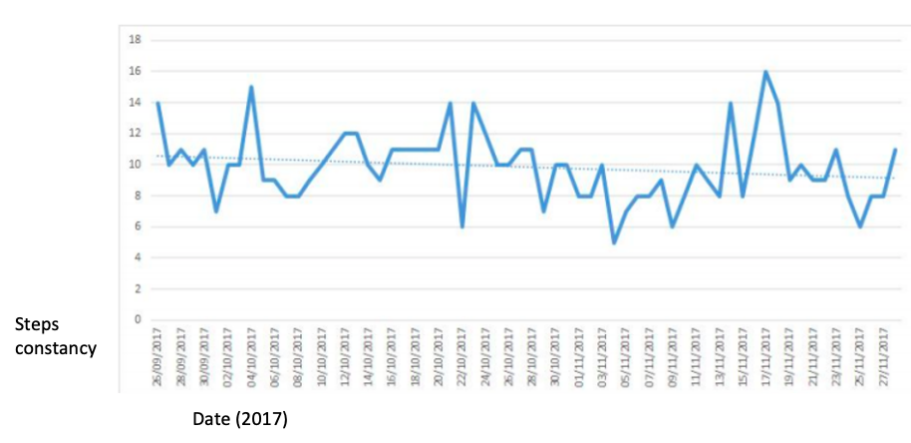
Daily steps constancy.

The evaluation of the study was aimed at investigating the effectiveness of the activity, the usability of the system, and the relevance of the organizational context in which the activity was organized. The first evaluation of the effectiveness of the activity was based on quantitative data: the average daily steps and the constancy (Figure 4) in doing 10,000 steps daily (x-axis: data and y-axis: number of steps). These data show a good level of engagement: the average daily steps per participant was 9660; the constancy oscillated between 4562 steps and 15,854 steps, as shown in the figure.

The second evaluation was based on qualitative data. The small number of participants involved in this first pilot study and the exploratory nature of the project led to the use of semistructured interviews and focus groups to collect the data. A total of 3 semistructured interviews were conducted with the operators and the coordinator of the center to examine in depth the characteristics of the context being studied and the perceptions of the stakeholders with regard to the project. The interviews had a duration of about an hour each. They were recorded and later transcribed. A total of 2 focus groups were used to involve the participants. The focus group is a particular type of focused meeting or interaction [37] that occurs when people recruited by a research group cooperate to turn their attention to a topic of discussion presented by a facilitator [38]. The reduced number of participants ensures and facilitates interaction [39]. Some authors [40] suggest the use of this technique to collect the opinions of older people on technology by stimulating a conversation about ideas or themes, bringing out important data that are difficult to investigate through face-to-face interviews [41]. The 2 focus groups involved 7 and 5 seniors. The first group discussion was attended by 3 members of a team and 4 members of the other team, a creator, and no team leader. The second group discussion saw the presence of 3 people from one team and 2 people from the other team, including the 2 team leaders. Both focus groups, which took place at the senior center, were led by a facilitator. The questions were asked to the whole group, and participants were encouraged to respond freely. The facilitator prepared a discussion guide with the questions (Table 2), which were considered “moments of an interaction ritual” [38] and therefore used flexibly and adapted to the conversation flow. Following an opening speech that described the plan for the meeting to the participants, they were asked questions about the organizational context of the project, the reasons that led them to participate, the usability of the technology, and the critical aspects of the gamification and finally some suggestions to improve the activity.

**Table 2:** Focus group discussion guide.

Stage	Question
Opening question	As you know, you have been asked to participate in a research project that looks at active ageing through games and the use of new technologies: today, I would like to discuss with you how the project went.
Introductory questions	How did you come across this project? (eg, flyer, word of mouth, operators’ invitation). What do you think about this project compared to other initiatives offered by the centre? Do you deem it appropriate for a centre to offer a larger number of initiatives involving physical exercise and technology?
Transitional questions	What were the reasons that led you to participate in the project? (eg, curiosity about technology, desire to take more exercise, company) Were there people who did not participate? Why?
Key questions	Since I was present only at some stages of the project, would you please tell me how the hunts worked? What suggestions would you give me in order to create interesting paths? (this question was made by the participant who created the path) Would you check your progress on the step counting app often? (eg, during the hunt, during day-to-day activities) What are, in your opinion, the reasons why you did not take pictures or make comments? Would you find yourselves checking on the progress of the other team on the website? And on the screenshot posted at the centre? Would you talk about it during the week? (Was there anyone who motivated the others? Would you talk about it only at the meetings at the centre?) What were the pros and cons of playing in a team? What suggestions would you give me in order to form teams that work well together?
Final question	Should we propose these activities in other contexts, and do you have any suggestions to improve it?

### Qualitative Data Analysis

With regard to the phase of transcription and analysis of the material collected during the focus groups, we adopted the *content analysis* or *template analysis* technique [4], a dynamic form of analysis oriented to summarizing the information content of data collected [42,43]. In particular, we opted for an *inductive* analysis of content, a “research method for the subjective interpretation of the content of text through the systematic classification process of coding and identifying themes or patterns” [44]. In the inductive analysis of the contents, categories derive directly from raw data: the use of prebuilt categories was therefore avoided [44-46]. The validity of the inference is ensured by a systematic codification process, which begins with the isolation of fragments of text by grouping them into common themes. For each topic, the different opinions of the participants were then identified, focusing on the ways in which they emerged and on the interactions with each other. In addition to the analysis of the verbal data content, the situational factors of the focus groups were considered [47], in particular, the interaction between the participants and the forms of social influence that occur in the group [38].

## Results

The inductive analysis of contents and interactions revealed 3 main categories: *gamifying self-tracking: motivation vs competition*, *gamifying geocaching: cooperation vs conflict*, and *gamifying active aging: empowerment vs assistance*. The categories have been illustrated in the following sections. For privacy reasons, we used fictitious names to report participant quotes.

### Gamifying Self-Tracking: From Motivation to Competition

A total of 3 factors motivated the elderly to participate in the project: curiosity about technology, the possibility of doing physical exercise with others, and an interest in cultural and historical aspects. From the analysis of the focus group conversations, the first aspect stands out: most of the participants stated that they were stimulated and intrigued by “using technology to support physical activity.” The pedometer was found to be the most motivating element for the entire duration of the project, among the technologies proposed (ie, smartphones with the C: geo app and a pedometer bracelet):

I kept checking how many steps I had taken and tried to increase them...when you can check and you realise that at some point you are almost there, then you go for a walk

Anna, participant

After having exercised regularly for a month, activity becomes a physical need

Gina, participant

Normally when you walk you don’t have the perception of taking so many steps

Sara, participant

You think you take so many steps at home...I never sit down...I take steps, but taking ten thousand...that’s a long way!

Diana, participant

The use of the pedometer and thus the possibility of keeping track of the steps taken were, therefore, critical to motivating the participants to carry out physical activity and to making them aware of their physical activity level. Between the lines, we could also read the *coercive* aspect of the pedometer, which for some has become an almost necessary control tool. This trend was confirmed by the fact that, although the project was finished, most of the focus group participants continued to wear and use the bracelets. It was apparent that this coercive aspect had positive effects on both physical well-being and personal satisfaction:

When you see that you have walked that much, you are happy

Gina, participant

...*and you have a result, because I feel better* [Sara, participant]

The possibility of keeping track of the steps had positive effects on the motivation of the individual participants to obtain results. In addition, the awareness that their steps would influence the score of their team transformed the pedometer into an instrument of competition and comparison with other participants:

Initially it was like a game, but then came the rivalry...when I saw that we were below the score, I started to do some serious walking...at ten o’clock in the evening I was around increasing our step levels...I had a share of score that I could not fail to achieve...I had to stay on that level...so it was challenging

Marina, participant

The expectations regarding the team scores were high. The meetings before the hunts turned into opportunities for scores to be compared, and taking steps was seen almost as an obligation: a participant who missed a day’s worth of walking to be present at a tournament of burraco (a card game) was (somewhat jokingly) accused of making their team lose. The creators of the hunt, despite not knowing which team they were assigned to, experienced this sense of challenge:

We didn’t even know which team we were helping, so it was a matter of constancy...then it almost became an obsession...I would check and say to myself “I have to reach ten thousand, otherwise they’ll hang me”

Rita, creator

The collective contribution to the group’s score led to a strong team spirit, which heightened the competition during the hunts, as can be seen from the following discussion:

For example, when we were solving a riddle, the others would come and we looked in the other direction to give them wrong clues...We didn't even say goodbye! That’s how much we felt we were enemies

Fiona, participant

I think the other team envied us...There was also some tipping off

Sara, participant

This strong sense of competition also emerged from the interaction between the focus group participants: opportunities for jokes, tensions, and even controversy between the members of the 2 teams were frequent.

### Gamifying Geocaching: From Cooperation to Conflict

As mentioned earlier, the possibility of counting the steps and contributing to the team’s score led to a strong sense of competition among the participants in the project. This sense of competition influenced the relations between individual participants and between the 2 teams and also involved other aspects of the gaming experience. All the focus group participants said that they were in a hurry to find all the caches:

We were mad...so we said “no comment”

Alda, participant

Also because often the riddle was not clear, but it needed to be understood

Fiona, participant

Did you focus on riddles or on the steps? Were we supposed to focus on taking many steps? As far as that is concerned, we succeeded...For the rest, I remember the hurry, but less hurry would have been nice for me since I don't know Trento...when the two teams met, people who knew each other didn’t even say goodbye

Marina, participant

Also, about entering our observations...how were we supposed to write something?...it would have been nice to be able to write down some nice comments

Margherita, participant

Owing to the rush to reach the points of interest before the opposing team, some peculiar features of the geocaching game slipped into the background, such as carefully reading the cache description, leaving a comment after finding the cache, or adding photographs. Interviews with operators during outings confirm this trend: “Due to the rush to move on fast, we did not focus on other smartphone features such as selfies or writing sentences” (Mario, social operator). The sense of competition led to the rush to participate in the game, to solve the riddles, and to comment below the found caches, and this led to a deviation from the initial motivation that was the interest in historical and cultural aspects. However, this did not happen in the case of the creators, who, having not experienced the sense of team competition, maintained their enthusiasm for these aspects:

Even now when I walk around the city I look up...it is actually by looking up that you discover things...I discovered, on a building, a coat of arms that I didn't even know existed

Gina, creator

The lack of interest in some features of the game should not be attributed solely to the sense of competition. In fact, some features of the technologies contributed to this situation: first, the decision to entrust the team leader with the map with the smartphone led to a series of difficulties:

It was backlit, too shiny, and it was sunny...it was difficult to mark the riddles solved...if there were a bigger screen, everyone would see it...if you could see the whole journey perfectly...if you zoom in, you lose the other piece

Alda, participant

One of the important points was to read the description of the hunt stop...I read it aloud during the journey because there was no time...but of course there was the one with the stick behind you who couldn’t hear

Margherita, participant

Hurdles were caused by the intrinsic features of the technology: the glare on the smartphone screen in the bright sun and a screen too small to display the whole map discouraged participants from using the tools necessary for carrying out the activity.

The comments reported so far have pointed out the ways in which some features of geocaching and aspects of gamification came into conflict, leading to hurdles that can become important when it comes to involving people of a certain age, as a participant stated in a very appropriate fashion:

If you want more people to have this chance, you should allow for much more time, including the chance to use the restroom, the chance to rest for those who walk with a stick...and to rest twice for those who feel they have walked too much...if instead you want to focus only on the treasure, then it is no longer for us

Margherita, participant

### Gamifying Active Aging: From Empowerment to Assistance

The development of the project saw the constant presence of the center operators, from the first presentations to the hunt coplanning with the creators, the treasure hunt, and the final awarding. During all these phases, their experiential contribution to the user experience proved critical, both in terms of organization and support with the use of technologies as well as the management of conflicts among participants. The organizational context proved essential for the involvement of the participants. On the one hand, the fact that the senior center operators were already accustomed to proposing activities of this kind and paying attention to technology and active aging translated into having a pool of people already interested in the subject and, above all, people who trusted the operators:

Ease of involvement: we know who comes here for technology-related concerns, about eighty people

Mario, social operator

Gina, who hadn’t been to the centre for a long time, was invited by Agnese, and came because she believed in it, trusted her

Cristina, social operator

On the other hand, the peculiarity of the center, owing to its recreational activities, allows the development of a strong network of relationships that, in turn, helps spread the word among people and facilitates chain involvement, thus allowing people to overcome the possible distrust of technologies:

The first time I didn’t know what it was, then you told me, “do you want to participate?”

Anna, participant

Many, when they saw that I was enthusiastic, wanted to participate

Rosa, participant

The intervention of the operators was critical to the success of the project, particularly when it came to supporting the elderly in the use of technologies:

The first hunt was chaotic...we struggled because even the two team leaders felt awkward...we [operators] also had to help a lot with the phones...but in the end we learned by doing.

Cristina, social operator

We were always supported by Mario and Cristina, because if it happened they would jam.

Marina, participant

The team members who didn’t have a smartphone weren’t interested in using it. Maybe this fact may have taken the responsibility off the others with the risk that they would just follow...So I would say: “Come on, come here, let’s look together where the points are” and there my role was that of regrouping them.

Cristina, social operator

The operators were able to support the participants in the use of the app, and their intervention proved to be decisive in directing the group’s attention to the technology. This curbed an issue caused by the intrinsic characteristics of the technology and the spirit of competition among the participants.

Finally, the intervention of the operators allowed them to mediate some relational issues that emerged during the course of the project, such as the strong spirit of competition:

We had to work a lot on the team: we would encourage them to come closer, listen, help find the solution, look carefully...team members were granted a good level of autonomy but we had to provide assistance anyway as finding an agreement was not so simple.

Mario, social operator

As pointed out by Mario, the operator, although his main role was to support independent action on the part of the participants, in some cases, his presence was considered almost an excuse to delegate some activities: “No, we didn’t take pictures, we were in a hurry...there was either Mario or Cristina [social operators] to do that” (Rosa, participant).

## Discussion

### Principal Findings

To the best of our knowledge, this is the first study on active aging that has combined elements of self-tracking and gamification with a geocaching activity. The success of the project, backed by the data on daily walk constancy of the participants and their satisfaction, is in line with the results of the studies that show how adding gamification to self-tracking can be a valid strategy to promote physical exercise among the elderly. However, through our study, we wanted to describe how the introduction of gamification elements required the inclusion of heterogeneous contextual and organizational elements that proved to be critical in determining the success of the project. Therefore, the study aimed to highlight how promoting active aging through technologies does not happen solely with the use of these tools but instead requires a wider process that involves a complex, articulated network.

### Technologies as Responses to the Limits?

A study by Vartiainen and Tuunanen [48] pointed out the importance of the *hedonistic* value of geocaching: instead of stimulating the participants’ sense of competition, the primary value remains to be the stronger sense of community that is aroused in geocachers. Our study aimed to propose a geocaching activity to promote active aging, integrated with elements of quantification (step counting) and gamification (team challenge). These elements—the pedometer bracelet, the team scoring mechanism, and the counting of the caches—proved to be highly motivating for the participants; however, they also led to a strong sense of competition, altering the nature of the activity and coming into conflict with some of the intrinsic characteristics of the game. For instance, in an endeavor to only come first, the participants did not use some features of the geocaching app, such as the ability to read cache descriptions, leave comments, and take photos. The haste and competition also led to some moments of tension and conflict among the participants. The correct use of the app and the mediation of conflicts could only take place thanks to the support of the center operators who mediated the conflicts and introduced older people to the use of technology. The technological tools were therefore integrated into a context that determined the methods of use: the familiarity of the social center with the technologies and the support of the social operators contributed to recreating an experience that was tailored to the skills and difficulties of the participants.

One of the most positive aspects of the project turned out to be the successful involvement and motivation of the participants. Although the involvement of users has become necessary for many technological innovations in different sectors [49], it has so far received little attention as an epistemic process itself. In most studies on active aging, there is an “accommodationist view” [50], which sees technologies designed as mere solutions to age-related needs and limitations. In our study, we wanted to go beyond this vision, focusing on the active involvement of the participants in every planning phase, from the conception of the paths to the presentation of the project to their peers. We wished to create a protagonism of elderly people, who do not feel abandoned but are accompanied by the caregivers on their journey toward autonomy and independence. This path is not exempt from contradictions, as is evident from the fact that the intervention of operators can also become an excuse to delegate the use of technology. However, this confirms that these limits derive from the interactions among players, institutions, and material objects [51]. As we highlighted in this paper, the introduction of technologies for active aging inevitably involves an interactive and evolving commitment with the social and cultural spheres of the users with all their difficulties, abilities, and constant reinventions.

### Study Limitations and Future Developments

Our study had some limitations and some lessons learned. The first limitation of the study concerned the limited nature of the sample examined. Although this was the first pilot study in which the qualitative methodologies proved to be most appropriate in achieving the research objectives, the project nevertheless took the first step toward the expansion to other organizations in the local area (such as senior centers, clubs, and schools), which would provide a larger sample. To this end, in the year following the pilot study described in this paper, the Impronte project saw 2 further additions: the first involved a school, in addition to the senior center, with a view to intergenerational exchange, and the second saw the inclusion of a natural park, with the objective of local seniors devising routes for tourists. The idea was to continue on this track, owing to the development of a geocaching platform designed by and for the elderly, which would allow heterogeneous and, in some cases, isolated contexts to be reached. A second limitation—and a lesson learned—of the study was the lack of a collection of pre- and postactivity data: analyzing the amount of steps taken by older people before and after participating in the project could provide interesting statistics on the motivation to maintain healthy lifestyles. Another lesson learned is with regard to encouraging participants to learn to use technology through geocaching. Our study provided that only 1 team component managed the phone during the game. This is because we had to decide whether, on the one hand, to encourage participants to learn to use technology, but with the risk that each participant would have to participate on his or her own and be isolated from the rest of the group, or whether, on the other hand, to encourage the social aspect of the game, stimulating the communication between participants. We chose the second option, but at the expense of encouragement to learn to use technology. It would be interesting to investigate if providing all participants with the opportunity to manage a phone would encourage them to learn to use technology.

### Conclusions

Activities in favor of active aging that involve the use of new technologies can foster the participation of elderly people, as was evident in the success of this project: an activity that allows outdoor exercise through a playful activity such as geocaching and the use of self-tracking tools can encourage and motivate older people to perform physical activity, as shown by the quantitative data. However, this paper aimed to show how technology use of older people was reshaped and contextualized during the project, with some aspects of the activity prioritized and others sent to the background.

It is important to go beyond the fact that users simply have to be involved and that the involvement of multiple users automatically leads to better knowledge. We propose that it is essential to develop a detailed understanding of how, when, and why they should be involved and also when and how they should not. Above all, users should actively participate in the creation of project contents from a peer-to-peer perspective, as was the case for the project described in this paper.

This approach can enable stakeholders, policy makers, and innovators to better understand the future of aging and address the challenges of innovation for older users more completely. Going beyond an *accommodating* vision that sees science and technology as mere solutions is critical to designing technologies that are functional for seniors (and their caregivers) and allow them to embrace the challenge of active aging.

## Abbreviations

APIs: application programming interfaces
